# Increased level and interferon-γ production of circulating natural killer cells in patients with scrub typhus

**DOI:** 10.1371/journal.pntd.0005815

**Published:** 2017-07-27

**Authors:** Seung-Ji Kang, Hye-Mi Jin, Young-Nan Cho, Seong Eun Kim, Uh Jin Kim, Kyung-Hwa Park, Hee-Chang Jang, Sook-In Jung, Seung-Jung Kee, Yong-Wook Park

**Affiliations:** 1 Department of Infectious Diseases, Chonnam National University Medical School and Hospital, Gwangju, Republic of Korea; 2 Department of Rheumatology, Chonnam National University Medical School and Hospital, Gwangju, Republic of Korea; 3 Department of Laboratory Medicine, Chonnam National University Medical School and Hospital, Gwangju, Republic of Korea; Mahidol University, THAILAND

## Abstract

**Background:**

Natural killer (NK) cells are essential immune cells against several pathogens. Not much is known regarding the roll of NK cells in *Orientia tsutsugamushi* infection. Thus, this study aims to determine the level, function, and clinical relevance of NK cells in patients with scrub typhus.

**Methodology/Principal findings:**

This study enrolled fifty-six scrub typhus patients and 56 health controls (HCs). The patients were divided into subgroups according to their disease severity. A flow cytometry measured NK cell level and function in peripheral blood. Circulating NK cell levels and CD69 expressions were significantly increased in scrub typhus patients. Increased NK cell levels reflected disease severity. In scrub typhus patients, tests showed their NK cells produced higher amounts of interferon (IFN)-γ after stimulation with interleukin (IL)-12 and IL-18 relative to those of HCs. Meanwhile, between scrub typhus patients and HCs, the cytotoxicity and degranulation of NK cells against K562 were comparable. CD69 expressions were recovered to the normal levels in the remission phase.

**Conclusions:**

This study shows that circulating NK cells are activated and numerically increased, and they produced more IFN-γ in scrub typhus patients.

## Author summary

*Orientia tsutsugamushi* is an obligate intracellular bacterium. It primarily invades endothelial cells, macrophages, monocytes, and dendritic cells. Plasma concentrations of interferon (IFN)-γ, several cytokines and chemokines, which are known to recruit natural killer (NK) cells and T cells, were found to be increased in scrub typhus patients. NK cells are known as essential immune cells against several pathogens. In murine models of *Rickettsial* infection, the clearance of bacteria was found to be significantly associated with NK cell activity. Not much is known regarding NK cells’ role in *O*. *tsutsugamushi* infection in humans. This study is very possibly the first to measure NK cells’ level and function of in scrub typhus patients, or to examine NK cell levels’ clinical relevance. This study’s results demonstrate that circulating NK cells are activated and numerically increased in scrub typhus patients. Notably, increased production IFN-γ by NK cells of scrub typhus patients suggests their contribution to enhancement of intracellular bacterial killing in infected antigen presenting cells. Moreover, disease severity corresponded to increased NK cell levels. These findings importantly suggest that NK cells play a role in protecting the host against *O*. *tsutsugamushi* infection.

## Introduction

*Orientia tsutsugamushi* is an obligate intracellular bacterium that causes scrub typhus in humans. It is a mite-borne, endothelium-targeting intracellular bacterium. Scrub typhus is prevalent in Asia, Northern Australia, and the Indian subcontinent. Most patients may recover from scrub typhus without complications if provided with an early diagnosis and management [1]. However, some patients develop fatal complications with median mortality of 6.0% unless they are treated sufficiently early in the course of illness [2]. *O*. *tsutsugamushi* resides in the cytoplasm of host cells, which are mainly endothelial cells, macrophages, monocytes and dendritic cells [3,4,5,6]. Related studies have found elevated plasma concentrations of interferon (IFN)-γ, IFN-γ-inducing cytokines (e.g., interleukin [IL]-12, IL-15, IL-18, and tumor necrosis factor [TNF]-α), and chemokines induced by IFN-γ (e.g., IFN-γ-inducible protein 10 and monokine induced by IFN-γ). These are well known for recruiting natural killer (NK) cells and T cells in patients with scrub typhus [7,8]. Based on these findings, a combination of innate and adaptive immune responses likely contribute to host defense against *O*. *tsutsugamushi*.

Natural killer (NK) cells are essential effectors within our innate immunity system. They mediate the elimination of target cells directly or indirectly through secretion of effector molecules such as perforin/granzyme, cytokines (mainly IFN-γ), and chemokines [9,10]. NK cells were discovered in the mid-1970s as they showed the ability to lyse tumor cells without prior exposure [11,12]. It is now well established that NK cells are also effective against several viruses, fungi, parasites and some intracellular bacteria such as *Salmonella*, *Listeria* and *Chlamydia* [10,13,14]. NK cell-mediated cytotoxicity is a complex process that involves receptor-mediated binding and signaling, synapse formation, granule polarization, and granule release [15]. Infection by intracellular pathogens leads to a decreased expression of major histocompatibility complex (MHC) class I antigens in host cells. This decrease reduces the infected host cell’s ability to interact with NK cells’ inhibitory receptors. In turn, the infected cell’s becomes more susceptible to lysis by NK cells, which leads to the destruction of the intracellular pathogen [16]. Similarly, K562 cells (which lack the MHC complex required to inhibit NK activity) are easily killed by NK cells. For this reason, these cells are often used for detection of NK cytotoxicity [17].

In murine models of *Rickettsial* infection, the clearance of bacteria was found to be significantly associated with NK cell activity and mice with NK cell deficiency showed increased susceptibility to infection [18]. However, studies have yet to explore the role of NK cells in *O*. *tsutsugamushi* infection in humans. Accordingly, this study aims to examine the level and function of NK cells in patients with scrub typhus, as well as the clinical relevance of NK cell levels.

## Methods

### Study subjects

The study cohort comprised 56 patients with scrub typhus (30 women and 26 men; mean age ± SD, 66.8 ± 13.0 years) and 56 age- and sex-matched healthy controls (HCs; 28 women and 28 men; mean age ± SD, 62.7 ± 8.03 years). Patients’ diagnoses required detecting *O*. *tsutsugamushi* antibodies via a passive hemagglutination assay (PHA) using Genedia Tsutsu PHA II test kits (GreenCross SangA, Yongin, Korea). Patients were positive for infection if test results showed a titer of ≥ 1:80 in a single serum sample, or at least a four-fold rise in antibody titer at a follow-up examination. According to the number of dysfunctional organs involved, severity of scrub typhus was classified into the following 3 grades as previously described [19,20]: mild disease (no organ dysfunction); moderate disease (one organ dysfunction); and severe disease (≥ 2 organ dysfunctions). Organ dysfunction is: (1) renal dysfunction, creatinine ≥ 2.5 mg/dL; (2) hepatic dysfunction, total bilirubin ≥ 2.5 mg/dL; (3) pulmonary dysfunction, bilateral pulmonary infiltration on chest X-rays with moderate to severe hypoxia (PaO2/FiO2 < 300 mmHg or PaO2 < 60 mmHg or SpO2 < 90%); (4) cardiovascular dysfunction, systolic blood pressure < 80 mmHg despite fluid resuscitation; and (5) central nervous system dysfunction, significantly altered sensorium with Glasgow Coma Scale (GCS) ≤ 8/15. All healthy controls were recruited in the Jeollanam-do area, which was the same area as the areas where the patients have developed. Controls had no history of autoimmune disease, infectious disease, malignancy, chronic liver or renal disease, diabetes mellitus, immunosuppressive therapy, or fever within 72 hours prior to enrollment.

### Ethics statement

The Institutional Review Board of Chonnam National University Hospital approved this study’s protocol. All participants provided written informed consent in accordance with the Declaration of Helsinki.

### Monoclonal antibodies (mAbs) and flow cytometry

This study used the following mAbs and reagents: Allophycocyanin (APC)-Cy7-conjugated anti-CD3, APC-conjugated anti-CD3, APC-conjugated anti-CD69, and fluorescein isothiocyanate (FITC)-conjugated anti-CD45, FITC-conjugated anti-CD56, FITC-conjugated anti-CD107a, FITC-conjugated anti-IFN-γ, phycoerythrin (PE)-conjugated anti-CD3, PE-conjugated anti-CD56, PE-conjugated anti-CD69, PE-Cy7-conjugated anti-TNF-α, PerCP-conjugated anti-CD3, PerCP-conjugated anti-CD45, FITC-conjugated mouse IgG isotype and PE-conjugated mouse IgG isotype control (all from Becton Dickinson, San Diego, CA). Cells were stained with combinations of appropriate mAb for 20 minutes at 4°C. Stained cells were analyzed on a Navios flow cytometer using Kaluza software (version 1.1; Beckman Coulter, Brea, CA).

### Isolation of peripheral blood mononuclear cells (PBMCs), NK cells, and CD69+ and CD69- NK cells

Peripheral venous blood samples were collected in heparin-containing tubes, and PBMCs were isolated by density-gradient centrifugation using Ficoll-Paque Plus solution (Amersham Biosciences, Uppsala, Sweden). NK cells were phenotypically identified as CD3-CD56+ cells by flow cytometry, as previously described [21,22]. NK cells were isolated using CD56 MicroBeads (Miltenyi Biotec, Bergisch Gladbach, Germany). The purity of CD3-CD56+ cells was greater than 95% as analyzed by flow cytometry. For sorting of CD69+ and CD69- NK cells, PBMCs were stained with APC-conjugated anti-CD3, FITC-conjugated anti-CD56, PerCP-conjugated anti-CD45, and PE-conjugated anti-CD69 mAb, and were sorted to obtain CD45+CD3-CD56+CD69+ and CD45+CD3-CD56+CD69- NK cells using a FACS Aria I sorter (BD Biosciences, Mountain View, CA) at purities of > 98%.

### Intracellular cytokine staining

IFN-γ and TNF-α expression in NK cells were detected by intracellular cytokine flow cytometry as previous described [20]. Briefly, freshly-isolated PBMCs (1 × 10^6^/well) were incubated in 1 mL complete media. The media consisted of RPMI 1640, 2 mM _L_-glutamine, 100 units/mL of penicillin, and 100 μg/mL of streptomycin. It was supplemented with 10% fetal bovine serum (FBS; Gibco BRL, Grand Island, NY). The incubation period was 24 hours in the presence of IL-12 (50 ng/mL; Miltenyi Biotec, Bergisch Gladbach, Germany) and IL-18 (50 ng/mL; Medical and Biological Laboratories, Woburn, MA). For intracellular cytokine staining, we added 10 μL of brefeldin A (GolgiPlug; BD Biosciences, San Diego, CA). The final concentration of brefeldin A was 10 μg/mL. After incubation for an additional four hours, cells were stained with APC-Cy7-conjugated anti-CD3, PE-conjugated anti-CD56 and APC-conjugated anti-CD69 mAbs for 20 minutes at 4°C, fixed in 4% paraformaldehyde for 15 minutes at room temperature, and permeabilized with Perm/Wash solution (BD Biosciences) for 10 minutes. Cells were then stained with FITC-conjugated anti-IFN-γ and PE-Cy7-conjugated anti-TNF-α mAbs for 30 minutes at 4°C and analyzed by flow cytometry.

### Cytotoxicities of PBMCs and purified NK cells

Isolated PBMCs or purified NK cells and K562 cells (CCL-243; ATCC) were used as effector and target cells, respectively. Cytotoxicities of PBMCs and NK cells were evaluated by flow cytometry at an effector-to-target (E:T) cell ratio of 20:1 and 4:1, respectively, as previously described [17,21]. Briefly, isolated PBMCs and purified NK cells were cocultured with K562 cells for 4 hours. Mixed effector and target cells were stained with FITC-conjugated anti-CD45 mAb for 20 minutes at 4°C, then washed once in phosphate buffered saline (PBS). They were afterwards resuspended in 0.5mL of PBS containing 20 μL of 1 μg/mL propidium iodide (Becton Dickinson), and then incubated for 15 minutes at room temperature. A flow cytometry determined the ratio of dead K562 cells.

### Luminex assay and enzyme-linked immunosorbent assay (ELISA)

Luminex assay was performed according to the manufacturer’s instructions. Briefly, 25 μL of each supernatant from Quantiferon tubes was thawed and analyzed undiluted to determine the concentrations of cytokines, including IFN-γ, IL-17, and TNF-α, by Luminex assay using Milliplex MAP human Cytokine/Chemokine panel (Millipore, Billerica, CA) on a Bio-Plex 200 system with Bio-Plex Manager Software (version 4.1.1; Bio-Rad, Hercules, CA). Plasma levels of IL-12p40 and IL-18 were measured using a commercially available ELISA kit (R&D Systems Inc, Minneapolis, MN) according to the manufacturer’s instructions.

### CD107a degranulation assay

Degranulation of NK cells in response to K562 cells was determined by flow cytometry as previously described [23,24,25]. Briefly, freshly isolated PBMCs were stained with FITC-conjugated anti-CD107a or isotype control mAb and then incubated with or without K562 cells at an E:T ratio of 20:1. After 1 hour, monensin (GolgiStop; BD Biosciences, San Diego, CA) and brefeldin A were added, and the cells were incubated for an additional 4 hours at 37°C in 5% CO2. After the incubation, the cells were stained with PerCP-conjugated anti-CD3 and PE-conjugated anti-CD56 mAb for 20 minutes at 4°C, fixed in 4% paraformaldehyde for 15 min at room temperature, and analyzed by flow cytometry.

### Statistical analysis

All comparisons of percentages and absolute numbers of NK cells, expression levels of CD69 and CD107a in NK cells, and cytotoxicity were performed by analysis of covariance after adjusting for age and sex using Bonferroni correction for multiple comparisons. Expression levels of IFN-γ and TNF-α in NK cells were analyzed using unpaired t-test. The Mann-Whiney U test was used to compare plasma levels of cytokines in scrub typhus patients versus age- and sex-matched HCs. Linear regression analysis tested associations between NK cell levels and clinical or laboratory parameters. A Wilcoxon matched-pairs signed rank test compared changes in NK cell levels and activation according to disease activity. *P* values less than 0.05 were considered statistically significant. SPSS version 18.0 software (SPSS, Chicago, IL) performed the statistical analysis. GraphPad Prism version 5.03 software (GraphPad Software, San Diego, CA) performed graphic works.

## Results

### Subject characteristics

The clinical and laboratory characteristics of scrub typhus patients are summarized in Table 1. A total of 56 scrub typhus patients were included in this study. According to disease severity criteria that is based on number of organ dysfunctions, 33 patients (58.9%) had mild disease; 14 patients (25%) had moderate disease; and 9 patients (16.1%) had severe disease.

**Table 1:** Clinical and laboratory characteristics of 56 scrub typhus patients.

Variables	Scrub typhus	
Age (years), mean ± SD	66.8 ± 13.0	
male/female, n	26/30	
Clinical variables, n (%)		
Fever	55	(98.2)
Rash	35	(62.5)
Eschar	45	(80.4)
Confusion	4	(7.1)
Severity of the disease, n (%)		
Mild disease	33	(58.9)
Moderate disease	14	(25.0)
Severe disease	9	(16.1)
Organ dysfunction, n (%)		
Renal dysfunction	4	(7.1)
Hepatic dysfunction	7	(12.5)
CNS dysfunction	2	(3.6)
Respiratory dysfunction	16	(28.6)
Circulatory dysfunction	7	(12.5)
Co-morbid conditions, n (%)		
Diabetes mellitus	16	(28.6)
Cardiovascular disease	3	(5.4)
Chronic kidney disease	3	(5.4)
Chronic hepatic disease	2	(3.6)
Malignancy	3	(5.4)
Laboratory variables, mean ± SD		
Leukocyte count (cells/μL)	8839 ± 5292	
Lymphocyte count (cells/μL)	1621 ± 1021	
Hemoglobin level (g/dL)	12.4 ± 1.7	
Neutrophil count (cells/μL)	6389 ± 4915	
Platelet count (×10^3^ cells/μL)	145 ± 62	
Total bilirubin level (mg/dL)	1.2 ± 1.2	
Total protein level (g/dL)	6.1 ± 0.7	
Albumin level (g/dL)	3.2 ± 0.6	
AST level (U/L)	113 ± 133	
ALT level (U/L)	100 ± 115	
Alkaline phosphatase level (U/L)	142 ± 115	
LDH level (U/L)	784 ± 240	
CRP level (mg/dL)	9.3 ± 7.0	
ESR level (mm/hour)	24.8 ± 24.2	
Time to hospital visit (days)^*^, mean ± SD	5.5 ± 0.5	

*Abbeviations*: ALT = alanine aminotransferase

AST = aspartate aminotransferase

CNS = central nervous system

CRP = C-reactive protein

ESR = erythrocyte sedimentation rate

LDH = lactate dehydrogenase

n = number

SD = standard deviation.

* indicates time from symptom onset to antibiotic therapy.

### Increased numbers of circulating NK cells in scrub typhus patients

**Fig 1:**
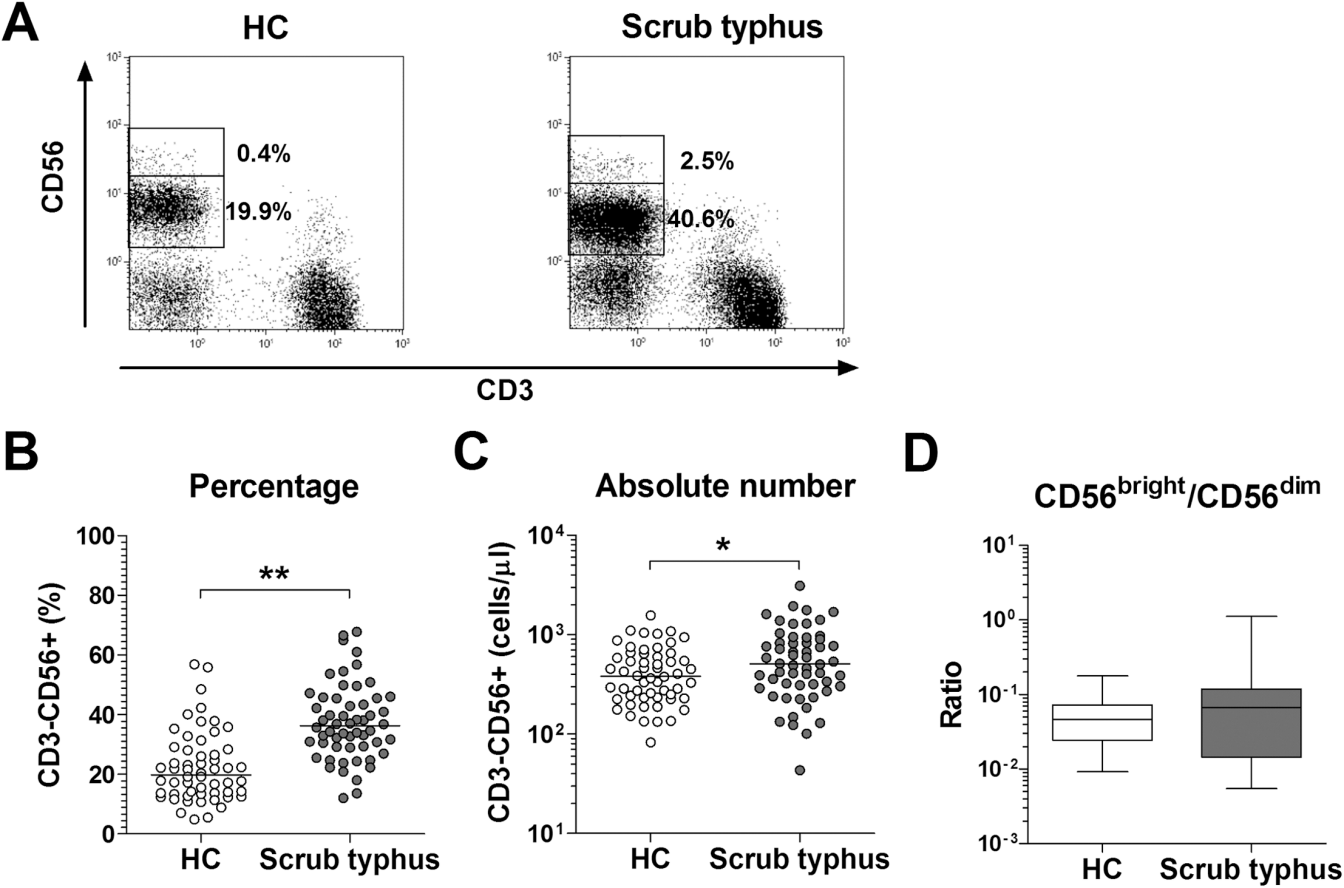
Increased circulating NK cell numbers in the peripheral blood of scrub typhus patients. Freshly isolated PBMCs from 56 HCs and 56 patients with scrub typhus were stained with FITC-conjugated anti-CD56, PE-conjugated anti-CD3, and PerCP-conjugated anti-CD45 mAbs, and then analyzed by flow cytometry. Percentages of NK cells were calculated using a CD45/SSC gate. Panel A: Representative NK cell percentages as determined by flow cytometry. Panel B: NK cell percentages among peripheral blood lymphocytes. Panel C: Absolute NK cell numbers (per microliter of blood). Symbols represent individual subjects and horizontal lines indicate median values. Panel D: The ratios of CD3-CD56^bright^/CD3-CD56^dim^ NK cell percentages among peripheral blood lymphocytes. Data are shown as box plots. Each box represents the 25th and 75th percentiles. The line inside the boxes represent the median. Whiskers represent the 10th and 90th percentiles. *p < 0.05, **p < 0.0005 by the ANCOVA test.

Flow cytometry determined the percentages and absolute numbers of NK cells in the peripheral blood samples of 56 patients with scrub typhus and 56 HCs. NK cells were defined as CD3-CD56+ cells (Fig 1A). Percentages of circulating NK cells were significantly higher in scrub typhus patients than in HCs (median 36.3% versus 19.8% [p < 0.0005]) (Fig 1B). Absolute numbers of NK cells were calculated by multiplying NK cell fractions by total lymphocyte numbers (per microliter of peripheral blood). Patients with scrub typhus had significantly higher absolute numbers of NK cells than HCs (median 507.6 cells/μL versus 380.8 cells/μL [p < 0.05]) (Fig 1C).

Based on the relative expression of the surface marker CD56, NK cells can be subdivided into CD56^bright^ and CD56^dim^ cells, as they exhibit different phenotypical and functional characteristics [26]. Previous studies have revealed that NK cell percentages, especially the proportion of CD56^dim^ NK cell subset, were increased in multiple organ failure syndrome after trauma and metastatic melanoma [27,28]. To determine whether increased NK cell numbers in scrub typhus were the consequence of the increased proportion of CD56^dim^ NK cell subset, the ratios of CD56^bright^ and CD56^dim^ NK cell subsets were measured by flow cytometry. No significant difference was observed in the ratio of CD56^bright^/CD56^dim^ NK cell subsets between scrub typhus patients and HCs (Fig 1D).

### Relationship between circulating NK cell levels and clinical parameters in scrub typhus patients

We used a regression analysis to investigate the correlation between NK cell percentages in the peripheral blood and other laboratory or clinical parameters (Table 2). The aim was to evaluate the clinical relevance of NK cell levels in 56 patients with scrub typhus. In the univariate linear regression analysis, circulating NK cell percentages positively correlated with age, leukocyte count, neutrophil count, and disease severity (p = 0.012, p = 0.001, p = 0.001, and p = 0.008, respectively). Meanwhile, circulating NK cell percentages inversely correlated with serum protein level and serum albumin level (p = 0.003 and p = 0.004, respectively). We observed no significant correlation between NK cell percentages and lymphocyte count, hemoglobin level, platelet count, total bilirubin level, aspartate aminotransferase level, lactate dehydrogenase level, C-reactive protein level, or the erythrocyte sedimentation rate (Table 2).

**Table 2:** Regression coefficients of NK cell percentages with respect to clinical and laboratory parameters in scrub typhus patients.

Variable	β	SE	p-value
Age (years)	0.323	0.124	0.012^*^
Leukocyte count (cells/μL)	0.001	0.001	0.001^*^
Lymphocyte count (cells/μL)	0.002	0.002	0.252
Hemoglobin level (g/dL)	-1.691	1.020	0.104
Neutrophil count (cells/μL)	0.001	0.001	0.001^*^
Platelet count (×10^3^ cells/μL)	0.016	0.029	0.578
Total bilirubin level (mg/dL)	0.960	1.439	0.507
Total protein level (g/dL)	-7.136	2.307	0.003^*^
Albumin level (g/dL)	-8.312	2.752	0.004^*^
AST level (U/L)	-0.002	0.013	0.859
ALT level (U/L)	-0.001	0.015	0.930
Alkaline phosphatase level (U/L)	0.019	0.015	0.203
LDH level (U/L)	0.007	0.007	0.372
CRP level (mg/dL)	0.487	0.246	0.053
ESR level (mm/hour)	0.041	0.122	0.741
Severity	5.864	2.115	0.008^*^

*Abbreviations*: ALT = alanine aminotransferase

AST = aspartate aminotransferase

β = regression coefficients

CRP = C-reactive protein

ESR = erythrocyte sedimentation rate

LDH = lactate dehydrogenase

SE = standard error.

* indicates statistical significance.

### Activation of circulating NK cells in scrub typhus patients

**Fig 2:**
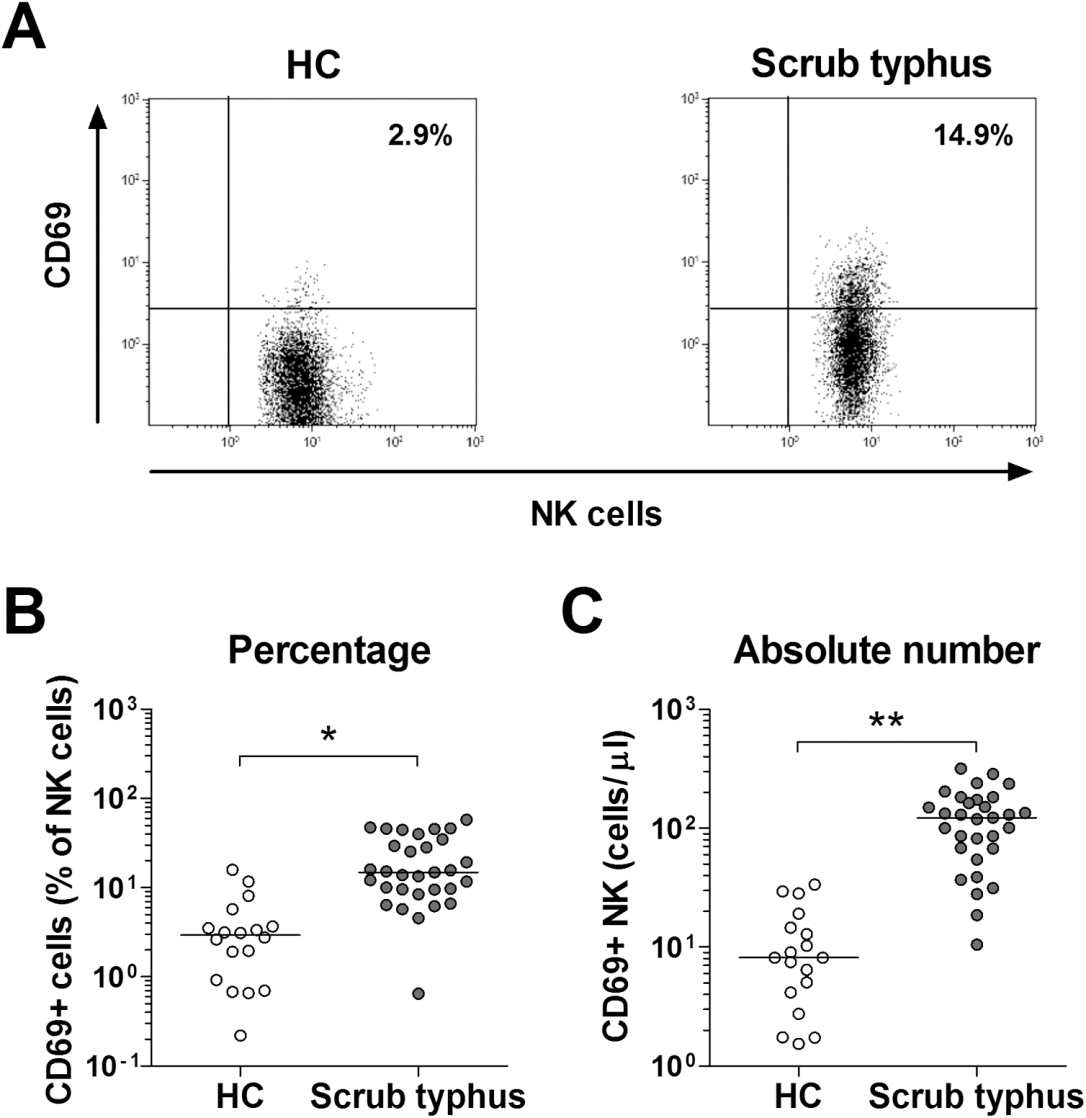
Expression of CD69 in circulating NK cells obtained from scrub typhus patients. Freshly isolated PBMCs from 18 HCs and 31 patients with scrub typhus were stained with FITC-conjugated anti-CD56, PE-conjugated anti-CD69, APC-conjugated anti-CD3, and PerCP-conjugated anti-CD45 mAbs, and then analyzed by flow cytometry. Panel A: Representative CD69-expressing NK cell percentages as determined by flow cytometry. Panel B: Percentages of CD69-expressing cells among NK cells. Panel C: Absolute CD69-expressing NK cell numbers (per microliter of blood). Symbols represent individual subjects and horizontal lines indicate median values. *p < 0.005, **p < 0.0001 by the ANCOVA test.

To determine whether NK cells were activated during infection by *O*. *tsutsugamushi*, we used flow cytometry to examine the expression of CD69 in circulating NK cells from 31 scrub typhus patients and 18 HCs. Percentages of CD69+ NK cells were significantly higher in scrub typhus patients than in HCs (median 14.9% versus 2.9% [p < 0.005]) (Fig 2A and 2B). Scrub typhus patients had significantly higher absolute numbers of CD69+ NK cells than HCs (median 122.7 cells/μL versus 8.2 cells/μL [p < 0.0001]) (Fig 2C).

### Increased plasma levels of IFN-γ and IFN-γ-inducing cytokines in scrub typhus patients

**Fig 3:**
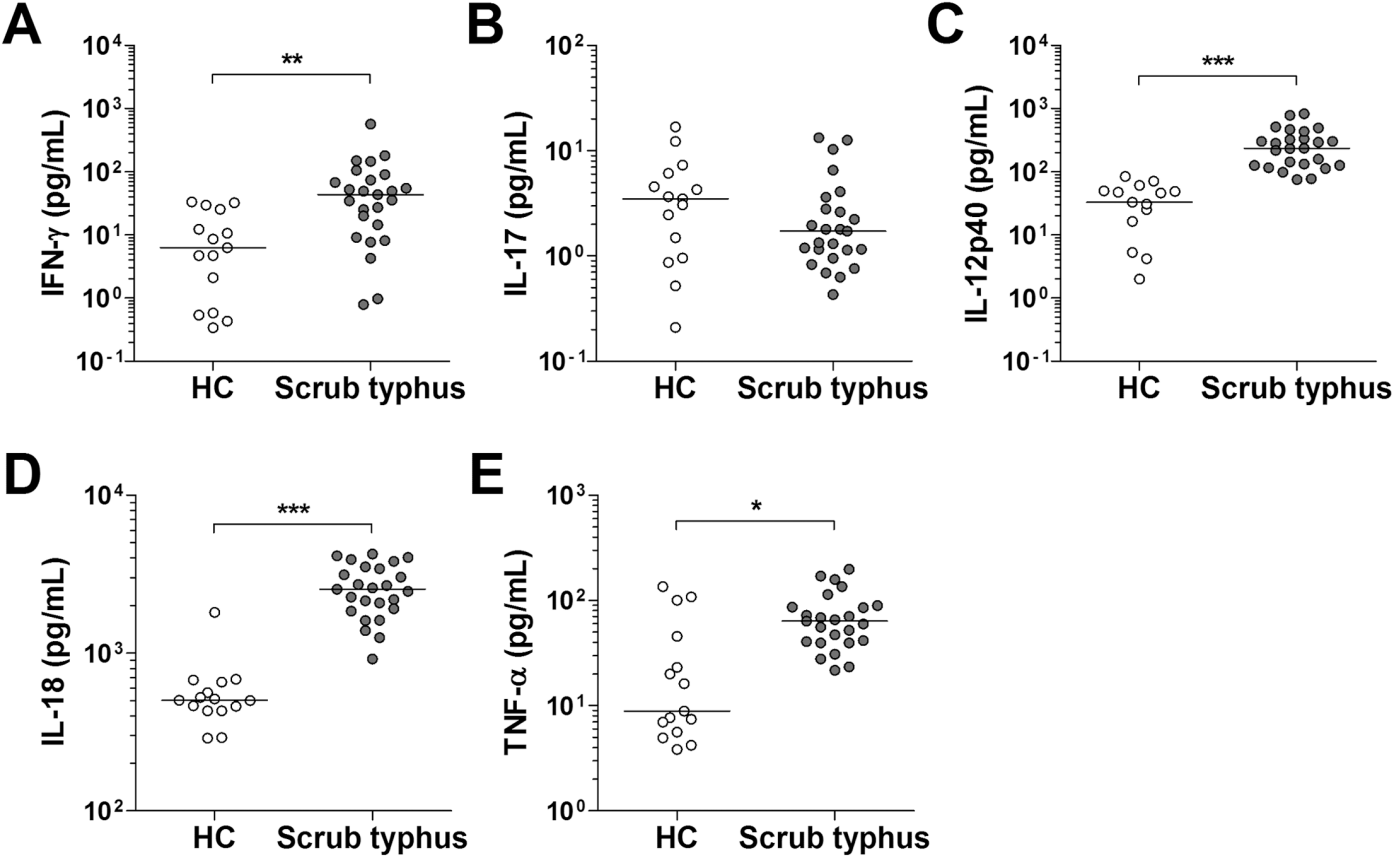
Plasma levels of IFN-γ, IL-12, IL-17, IL-18 and TNF-α in patients with scrub typhus. Plasma samples of the patients were collected before specific treatment on admission. Plasma levels of IFN-γ (panel A), IL-17 (panel B), IL-12 (panel C), IL-18 (panel D), and TNF-α (panel E) were determined by Luminex and ELISA. Data were obtained from 15 age- and sex-matched HCs and 25 patients with scrub typhus. Symbols represent individual subjects and horizontal lines indicate median values. *p < 0.005, **p < 0.001, ***p < 0.0001 by the Mann-Whitney U test.

A variety of cytokines can regulate NK cell functions, including activation, cytokine release, and cytotoxicity. Next, we measured plasma levels of IFN-γ, IL-17, and IFN-γ-inducing cytokines, such as IL-12, IL-18 and TNF-α, in 25 patients with scrub typhus and 15 age- and sex-matched HCs using Luminex assay and ELISA. Scrub typhus patients had significantly higher plasma IFN-γ levels than HCs (median 43.8 pg/mL versus 6.3 pg/mL [p < 0.001]) (Fig 3A). Plasma levels of all IFN-γ-inducing cytokines, including IL-12, IL-18, and TNF-α, were found to be significantly higher in scrub typhus patients than in HCs (medians: 236.4 pg/mL versus 33.1 pg/mL [p < 0.0001]; 2536 pg/mL versus 503 pg/mL [p < 0.0001]; and 63.8 pg/mL versus 8.9 pg/mL [p < 0.005], respectively) (Fig 3C, 3D and 3E). However, plasma IL-17 levels were comparable between scrub typhus patients and HCs (Fig 3B).

### Enhanced cytokine-mediated IFN-γ expression in NK cells of scrub typhus patients

**Fig 4:**
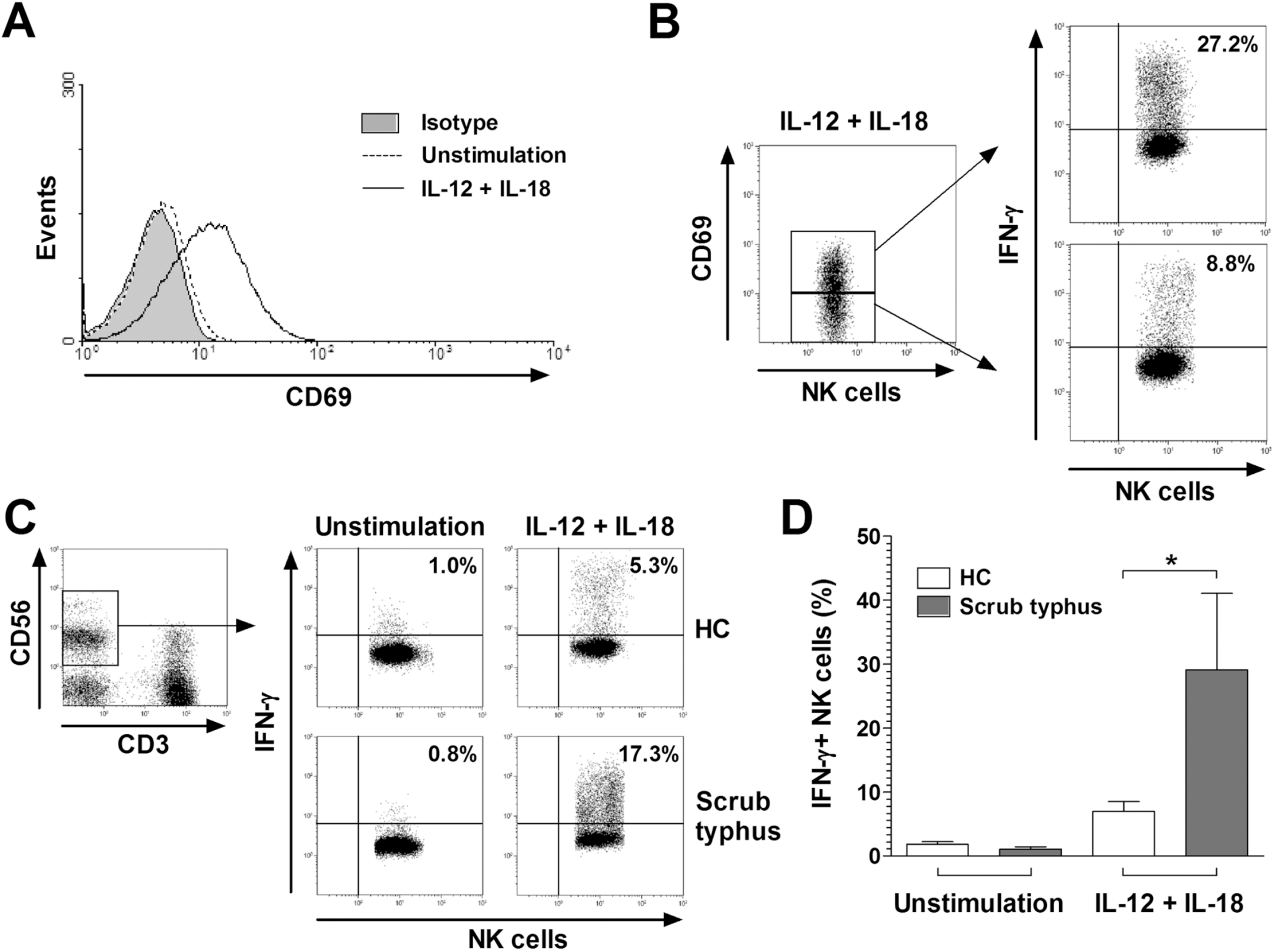
Cytokine-mediated CD69 and IFN-γ expression in NK cells. Freshly isolated PBMCs (1 × 10^6^/well) were incubated for 24 hours in the presence of IL-12 (50 ng/mL) and IL-18 (50 ng/mL), or PBS as a control. Expression levels of CD69 and IFN-γ in NK cells were determined by flow cytometry after stimulation with IL-12 and IL-18. Panel A: Representative CD69 expression in NK cells. Black line and dotted line represent CD69 expression in NK cells in the presence of IL-12 and IL-18 or PBS, respectively. Shaded regions indicate isotype-matched control mAbs. Panel B: Representative IFN-γ percentages in CD69+ and CD69- NK cell subsets. Data in panels A and B were obtained from 3 HCs. Panel C: Representative IFN-γ percentages in NK cells as determined by intracellular flow cytometry. Data in panel D were obtained from 10 HCs and 5 patients with scrub typhus. Values are expressed as the mean ± SEM. *p < 0.05 by unpaired t-test.

Based on our observation that IFN-γ-inducing cytokines were increased in scrub typhus patients, we next examined whether NK cells might be activated after stimulation with IL-12 and IL-18. We cultured PBMCs obtained from 3 HCs with IL-12 and IL-18 for 24 hours. We then determined the CD69+ cell levels in NK cells by flow cytometry. Percentages of CD69+ NK cells were significantly higher in IL-12- and IL-18-treated cultures than in untreated cultures (mean ± SEM 26.2 ± 4.6% versus 2.2 ± 0.6% [p < 0.05]) (Fig 4A and S1A Fig). Furthermore, to determine whether the production of IFN-γ by NK cells might be linked to the activation of NK cells, we measured the expression of IFN-γ in the CD69+ and CD69- NK cell populations of HCs in the presence of IL-12 and IL-18 for 24 hours at the single-cell level by intracellular cytokine flow cytometry. Percentages of IFN-γ+ NK cells were found to be significantly higher in CD69+ NK cell population than in CD69- NK cell population (mean ± SEM 25.5 ± 3.2% versus 12.6 ± 3.8% [p < 0.05]) (Fig 4B and S1B Fig).

NK cells are a critical component of the innate immune response because of their capacity to produce a variety of cytokines. Among the most prominent cytokines produced by NK cells are IFN-γ and TNF-α [29]. To investigate the expression of these cytokines in NK cells, we incubated PBMCs obtained from five patients with scrub typhus and 10 HCs for 24 hours in the presence of IL-12 and IL-18. We then examined the expressions of IFN-γ and TNF-α in the NK cell populations at the single-cell level by intracellular cytokine flow cytometry. Percentages of IFN-γ+ NK cells were higher in scrub typhus patients than in HCs (mean ± SEM 29.1 ± 12.0% versus 6.9 ± 1.6% [p < 0.05]) (Fig 4C and 4D). Similar results were obtained even in an experiment calculated by the absolute numbers of IFN-γ+ NK cells (mean ± SEM 125.3 ± 65.3 cells/μl versus 14.2 ± 5.2 cells/μl [p < 0.05]) (S2 Fig). However, all of the scrub typhus patients and HCs exhibited low levels of TNF-α+ NK cells, which were comparable between the two groups (S3 Fig).

### Cytotoxicity of NK cells in scrub typhus patients

**Fig 5:**
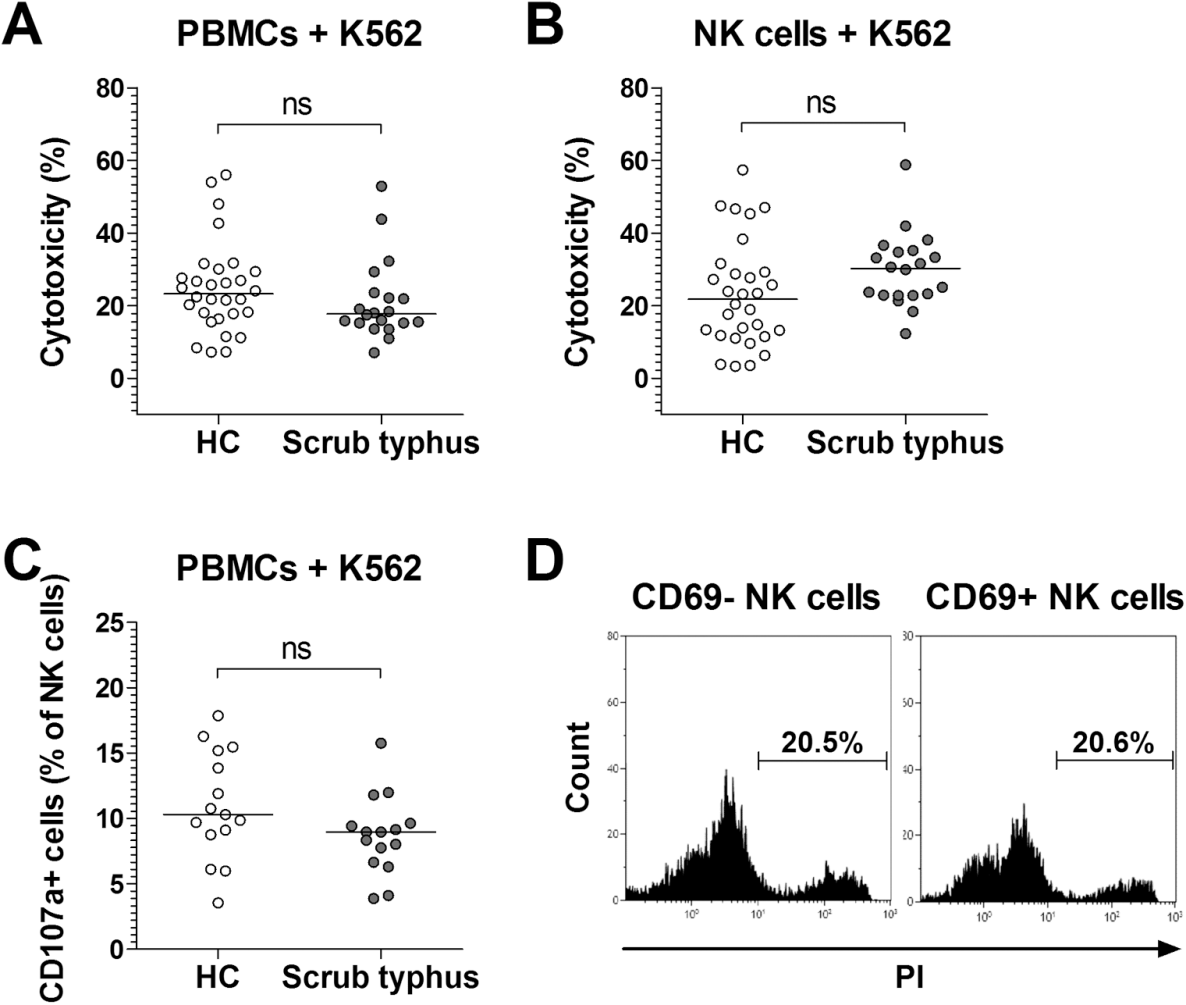
Cytotoxicity of NK cells in scrub typhus patients. Panel A and B: NK cytotoxicity. Freshly isolated PBMCs (panel A) or purified NK cells (panel B) from 30 HCs and 20 patients with scrub typhus were cocultured with K562 cells for 4 hours, and then stained with FITC-conjugated anti-CD45 mAb and PI. Cytotoxicity was determined as the percentage of apoptotic K562 cells by flow cytometry. Panel C: CD107a expression in NK cells. PBMCs obtained from 15 HCs and 15 patients with scrub typhus patients were stained with FITC-conjugated anti-CD107a or isotype control mAbs and then incubated with K562 cells. After 1 hour, monensin was added and the cells were incubated for an additional 4 hours. The cells were then stained with PerCP-conjugated anti-CD3 and PE-conjugated anti-CD56 mAbs, and then CD107a expression in NK cells was analyzed by flow cytometry. Symbols represent individual subjects and horizontal lines indicate median values. ns = not significant by the ANCOVA test. Panel D: NK cell cytotoxicity of purified CD69- and CD69+ NK cell subsets in scrub typhus patients. Results are representative of 3 independent experiments.

To examine the cytotoxic effect of NK cells on K562 cells, we used PBMCs and purified NK cells obtained from 20 patients with scrub typhus and 30 HCs. Cytotoxicities of PBMCs and purified NK cells were evaluated by flow cytometry at an effector-to-target (E:T) cell ratio of 20:1 and 4:1, respectively. The cytotoxicities were found to be comparable between scrub typhus patients and HCs (Fig 5A and 5B). Upon stimulation with K562 cells, the CD107a expression in NK cells was also comparable between scrub typhus patients and HCs (Fig 5C). Based on our observation that the expression level of IFN-γ was higher in CD69+ NK cells than in CD69- NK cells, we hypothesized that CD69+ NK cells could have an enhanced capacity to kill K562 cells. Thus, we determined cytotoxicities of purified CD69- and CD69+ NK cell subsets in scrub typhus patients by flow cytometry. However, we observed no significant difference in cytotoxicity between CD69- and CD69+ NK cell subsets in scrub typhus patients (Fig 5D).

### Changes in NK cell levels and CD 69 expression in scrub typhus patients according to disease activity

**Fig 6:**
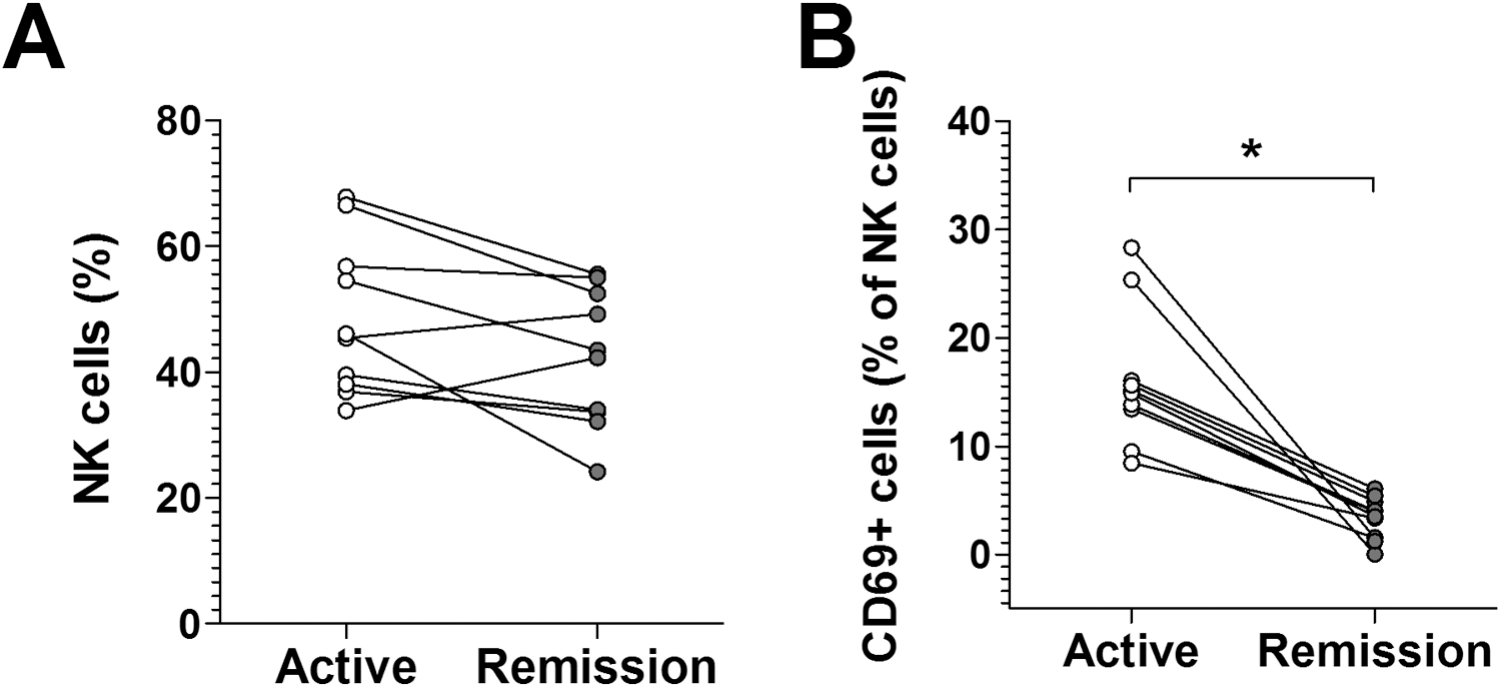
Changes in NK cell levels and CD 69 expression in scrub typhus patients. The percentages of NK cells (panel A) and CD69-expressing NK cells (panel B) in the peripheral blood of 11 scrub typhus patients during active disease and remission were determined by flow cytometry. Symbols represent individual subjects. *p < 0.005 by the Wilcoxon matched-pairs signed rank test.

We observed that circulating NK cell levels and CD69 expressions increased in scrub typhus patients; thus, we sought to determine circulating NK cell levels and CD69 expressions in the active and remission phases of the illness. Active and remission phases were defined as the period from the onset of symptoms to the start of antibiotic therapy on admission and the resolution of all presenting symptoms of scrub typhus after antibiotic treatment, respectively. Eleven scrub typhus patients were available for follow-up examination. As shown in Fig 6A, no significant changes in NK cell levels were found according to disease activity. However, CD69 expression was found to be significantly reduced when the disease was in remission than when it was active (median 3.5% versus 15.1% [p < 0.005]) (Fig 6B).

## Discussion

This is the first study to measure the level and function of NK cells in scrub typhus patients and to examine the clinical relevance of NK cell levels. The present study showed that circulating NK cell levels, together with elevated expression levels of CD69 and IFN-γ, increased in scrub typhus patients. Increased percentages of circulating NK cells reflected disease severity. Moreover, the plasma levels of IFN-γ, IL-12, IL-18 and TNF-α were significantly higher in scrub typhus patients than in HCs. In particular, with stimulation of NK cells with IFN-γ-inducing cytokines (i.e., IL-12 and IL-18), CD69 expression in NK cells was found to be increased, and CD69+ NK cells produced more IFN-γ than CD69- NK cells. Elevated CD69 expression in the active phase was normalized in the remission phase. However, the cytotoxicity and degranulation ability of NK cells were comparable between scrub typhus patients and HCs. Taken together, these findings suggest that cytokine-mediated activated NK cells accelerate the production of IFN-γ in scrub typhus patients.

Our results showed that percentages and absolute numbers of total NK cells in peripheral blood increased in scrub typhus patients. This result is substantiated by previous studies that found increased circulating NK cell levels in various human acute viral infections [30,31,32], whereas NK cell levels in peripheral blood have been reported to be decreased in influenza or HBV infection [33,34]. However, little is known about the circulating NK cell levels in human bacterial infection as compared with those in viral infection. Giamarellos-Bourboulis et al. have reported increased circulating NK cell levels in Gram-negative severe sepsis in humans [35], whereas others have reported a decline in NK cells in severe sepsis or septic shock [36,37,38]. Interestingly, only one study reported no evidence of change in the frequency of total NK cells in scrub typhus patients [39]. These discrepant findings about circulating NK cell levels might be due to the differences in pathogen, severity, time point at which the sample was obtained, and cohort selection bias including age and sex studied. In particular, age and sex are well-known confounding factors affecting NK cell levels in humans [40,41]. In the present study, comparative analyses showed that NK cell levels were compensated by age and sex, and that NK cell levels were still significantly higher in scrub typhus patients. Furthermore, elevated NK cell numbers have been found to be a direct consequence of induced proliferation in humans infected with hantavirus [31]. When considered together, these findings suggest that the numerical increase in NK cells might be due to NK cells proliferating in response to *O*. *tsutsugamushi* infection. Further study would be needed to confirm whether increased NK cell numbers are due to proliferation or recruitment to the blood.

Our data showed that CD69 expression and IFN-γ production in circulating NK cells were increased in *O*. *tsutsugamushi* infection, consistent with previous studies using several intracellular bacterial pathogens, including *Rickettsia conorii*, *Haemophilus ducreyi*, *Salmonella typhimurium*, *Mycobacterium tuberculosis*, *M*. *bovis* BCG, and *Listeria monocytogenes* [18,29,42,43,44]. It has been relatively well established that NK cell activation is closely related to cellular crosstalk with APCs such as dendritic cells (DCs) and macrophages as well as endothelium stimulated or infected by bacterial pathogens [29]. Based on the previous mechanistic study on NK cell activation by *Haemophilus ducreyi* [42], we speculated that *O*. *tsutsugamushi*-infected APCs possibly promote activation and IFN-γ production of NK cells through secretion of IL-12 and IL-18. The notion is supported by our observation that plasma levels of IL-12 and IL-18 were increased in scrub typhus patients and that addition of IL-12 and IL-18 upregulated CD69 expression in NK cells. Further study is required to determine whether APCs and NK cells form conjugates during cocultures with *O*. *tsutsugamushi*. In addition, Kang et al. have reported that the induced production of IFN-γ in NK cells affected the production of inducible nitric oxide synthase (iNOS) in APCs [45], which might contribute to intracellular bacterial killing in *Listeria* infection. Collectively, these findings suggest that increased production of IFN-γ by activated NK cells via cellular crosstalk with *O*. *tsutsugamushi*-infected APCs contribute to intracellular bacterial killing in scrub typhus.

NK cells kill virally-infected or malignant-host cells by degranulation of granzymes and perforin [46,47]. In several previous studies, cytotoxicity of NK cells against bacteria-infected APCs was found to be increased [18,43]. Our data showed that cytotoxicity and degranulation of NK cells against malignant cells (e.g., K562) were comparable between scrub typhus patients and HCs, suggesting that *O*. *tsutsugamushi* infection does not enhance the cytotoxic effects of NK cells.

We observed a positive correlation between circulating NK cell levels and age, leukocyte count, neutrophil count, and disease severity. This positive correlation has also been described in Epstein-Barr virus infection [48], whereas a negative correlation has been reported in influenza [49]. As shown in S1 Table, the univariate linear regression analysis showed that the absolute numbers of total NK cells were also positively correlated with leukocyte count and lymphocyte count (p = 0.008 and p = 0.001, respectively), but lost its correlation with age, neutrophil count, total protein level, albumin level, and severity. However, little is known about the dynamics of circulating NK cells related to disease severity in bacterial infection as compared with viral infection. Interestingly, CD69+ NK cell levels were also found to be positively correlated with leukocyte count, neutrophil count, and disease severity (S2 Table and S4 Fig). Taken together, these findings indicate that the activated subset rather than total NK cells contribute more to the correlation between the absolute numbers of NK cells and severity or inflammatory parameters in scrub typhus. Considering the previous observation obtained using animal models that overzealous activation of NK cells was related to organ damage, regardless of the infection itself [50,51], there is a possibility that an increased level of NK cells might be a cause of severe scrub typhus infection. However, this notion was contradicted by our data which demonstrated that increased CD69+ NK cell levels in the acute phase of scrub typhus infection were recovered to the normal levels in the remission phase, implicating that NK cell activation and expansion might be a consequence of severe scrub typhus infection.

In this study, increase in NK cell number levels did not change over our observation time, which was a relatively short time interval. This result is consistent with data from our previous study on mucosal-associated invariant T cell levels. In that previous study, mucosal-associated invariant T cell levels also did not change over the same observed time period [20]. A long-term follow-up study could determine whether circulating NK cell levels recover to normal levels after scrub typhus infection. Such a follow-up study would be in line with other previous studies, which found that normalization of NK cell levels in acute viral infections took a relatively long time [48,49].

In summary, the present study demonstrates that circulating NK cells are activated and numerically increased, and they produced more IFN-γ in patients with scrub typhus. In addition, increased NK cell levels reflect disease severity.

## Supporting information

S1 Checklist STROBE checklist. (DOC)

S1 Table Regression coefficients of absolute NK cell numbers with respect to clinical and laboratory parameters in scrub typhus patients. (DOC)

S2 Table Regression coefficients of log-transformed absolute CD69+ NK cell numbers with respect to clinical and laboratory parameters in 31 scrub typhus patients. (DOC)

S1 Fig Cytokine-mediated CD69 and IFN-γ expression in NK cells. Freshly isolated PBMCs (1 × 10^6^/well) were incubated for 24 hours in the presence of IL-12 (50 ng/mL) and IL-18 (50 ng/mL), or PBS as a control. Expression levels of CD69 and IFN-γ in NK cells were determined by flow cytometry after stimulation with IL-12 and IL-18. Panel A: CD69 expression in NK cells derived from 3 independent experiments using HC as determined by flow cytometry. Panel B: IFN-γ-expressing cells in CD69+ and CD69- NK cell subsets derived from 3 independent experiments using HC as determined by intracellular flow cytometry. Values are expressed as the mean ± SEM. *p < 0.05 by paired t-test.
(TIF)

S2 Fig Cytokine-mediated IFN-γ expression of NK cells in patients with scrub typhus. Freshly isolated PBMCs (1 × 10^6^/well) were incubated for 24 hours in the presence of IL-12 (50 ng/mL) and IL-18 (50 ng/mL), or PBS as a control. Percentages of total and IFN-γ+ NK cells were determine by flow cytometry among lymphocytes and total NK cell proportion. Absolute IFN-γ+ NK cell numbers were calculated by multiplying NK cell and IFN-γ+ NK cell percentages by total lymphocyte numbers (per microliter). Data were obtained from 10 HCs and 5 patients with scrub typhus. Values are expressed as the mean ± SEM. *p < 0.05 by unpaired t-test.
(TIF)

S3 Fig Expression of TNF-α by NK cells from scrub typhus patients. PBMCs (1 × 10^6^/well) were incubated for 24 hours in the presence of IL-12 (50 ng/mL) and IL-18 (50 ng/mL), or PBS as a control. Expressions of TNF-α in NK cells were determine by flow cytometry after stimulation with IL-12 and IL-18. Percentage of TNF-α-expressing cells in total NK cells as determined by intracellular flow cytometry. Data were obtained from 10 HCs and 5 scrub typhus patients. Values are expressed as the mean ± SEM. An unpaired t-test was used to compare expression levels of TNF-α in scrub typhus patients versus healthy controls.
(TIF)

S4 Fig Circulating NK cell levels in patients with scrub typhus according to the disease severity. Freshly isolated PBMCs from patients with scrub typhus were stained with either a cocktail of FITC-conjugated anti-CD56, PE-conjugated anti-CD3, and PerCP-conjugated anti-CD45 mAbs or another cocktail of FITC-conjugated anti-CD56, PE-conjugated anti-CD69, APC-conjugated anti-CD3, and PerCP-conjugated anti-CD45 mAbs, and then analyzed by flow cytometry. According to the disease severity, scrub typhus are classified into mild, moderate, and severe disease. Panel A and B: Percentages (among peripheral blood lymphocytes) and absolute numbers (per microliter of blood) of total NK cell population for a total of 56 patients with scrub typhus. Panel C and D: Percentages (among total NK cells) and absolute numbers (per microliter of blood) of CD69+ NK cell population for a total of 31 patients with scrub typhus. Symbols represent individual subjects and horizontal lines indicate median values. The p values for the trend of NK cell levels according to the disease severity were calculated by the Jonckheere-Terpstra test.
(TIF)
