# Supplementary material for: Increased level and interferon-γ production of circulating natural killer cells in patients with scrub typhus
Source: PLoS Negl Trop Dis. 2017 Jul 27;11(7):e0005815. doi: 10.1371/journal.pntd.0005815 (PMC5549767; doi:10.1371/journal.pntd.0005815)
Supplement: S1 Table — (DOC) [file pntd.0005815.s002.doc]

**S1 Table. Regression coefficients of absolute NK cell numbers with respect to clinical and laboratory parameters in scrub typhus patients.**

| Variable | β | SE | p-value |
| --- | --- | --- | --- |
| Age (years) | -0.075 | 5.884 | 0.990 |
| Leukocyte count (cells/μL) | 0.039 | 0.014 | 0.008^*^ |
| Lymphocyte count (cells/μL) | 0.348 | 0.061 | 0.001^*^ |
| Hemoglobin level (g/dL) | -19.19 | 46.83 | 0.684 |
| Neutrophil count (cells/μL) | 0.027 | 0.016 | 0.090 |
| Platelet count (×10^3^ cells/μL) | 1.122 | 1.273 | 0.382 |
| Total bilirubin level (mg/dL) | 15.97 | 64.93 | 0.807 |
| Total protein level (g/dL) | -98.22 | 111.6 | 0.383 |
| Albumin level (g/dL) | -143.4 | 131.4 | 0.280 |
| AST level (U/L) | -0.083 | 0.576 | 0.886 |
| ALT level (U/L) | -0.047 | 0.667 | 0.944 |
| Alkaline phosphatase level (U/L) | 0.690 | 0.662 | 0.302 |
| LDH level (U/L) | 0.320 | 0.345 | 0.359 |
| CRP level (mg/dL) | 15.14 | 11.25 | 0.184 |
| ESR level (mm/hour) | -3.341 | 6.659 | 0.622 |
| Severity | 68.33 | 100.5 | 0.500 |

*Abbreviations*: ALT = alanine aminotransferase; AST = aspartate aminotransferase; β = regression coefficients; CRP = C-reactive protein; ESR = erythrocyte sedimentation rate; LDH = lactate dehydrogenase; SE = standard error.

*indicates statistical significance.
