# Supplementary material for: Increased level and interferon-γ production of circulating natural killer cells in patients with scrub typhus
Source: PLoS Negl Trop Dis. 2017 Jul 27;11(7):e0005815. doi: 10.1371/journal.pntd.0005815 (PMC5549767; doi:10.1371/journal.pntd.0005815)
Supplement: S2 Table — (DOC) [file pntd.0005815.s003.doc]

**S2 Table. Regression coefficients of log-transformed absolute CD69+ NK cell numbers with respect to clinical and laboratory parameters in 31 scrub typhus patients.**

| Variable | β | SE | p-value |
| --- | --- | --- | --- |
| Age (years) | <0.001 | 0.006 | 0.983 |
| Leukocyte count (cells/μL) | <0.001 | <0.001 | 0.039^*^ |
| Lymphocyte count (cells/μL) | <0.001 | <0.001 | 0.811 |
| Hemoglobin level (g/dL) | -0.006 | 0.048 | 0.894 |
| Neutrophil count (cells/μL) | <0.001 | <0.001 | 0.040^*^ |
| Platelet count (×10^3^ cells/μL) | 0.001 | 0.001 | 0.423 |
| Total bilirubin level (mg/dL) | 0.154 | 0.070 | 0.036^*^ |
| Total protein level (g/dL) | -0.092 | 0.092 | 0.325 |
| Albumin level (g/dL) | -0.298 | 0.120 | 0.019^*^ |
| AST level (U/L) | <0.001 | 0.001 | 0.841 |
| ALT level (U/L) | <0.001 | 0.001 | 0.571 |
| Alkaline phosphatase level (U/L) | <0.001 | 0.001 | 0.359 |
| LDH level (U/L) | <0.001 | <0.001 | 0.591 |
| ESR level (mm/hour) | 0.020 | 0.012 | 0.251 |
| CRP level (mg/dL) | 0.017 | 0.011 | 0.152 |
| Severity | 0.194 | 0.076 | 0.016^*^ |

*Abbreviations*: ALT = alanine aminotransferase; AST = aspartate aminotransferase; β = regression coefficients; CRP = C-reactive protein; ESR = erythrocyte sedimentation rate; LDH = lactate dehydrogenase; SE = standard error.

*Indicates statistical significance.
