# Supplementary figures and images for: Increased level and interferon-γ production of circulating natural killer cells in patients with scrub typhus
Source: PLoS Negl Trop Dis. 2017 Jul 27;11(7):e0005815. doi: 10.1371/journal.pntd.0005815 (PMC5549767; doi:10.1371/journal.pntd.0005815)

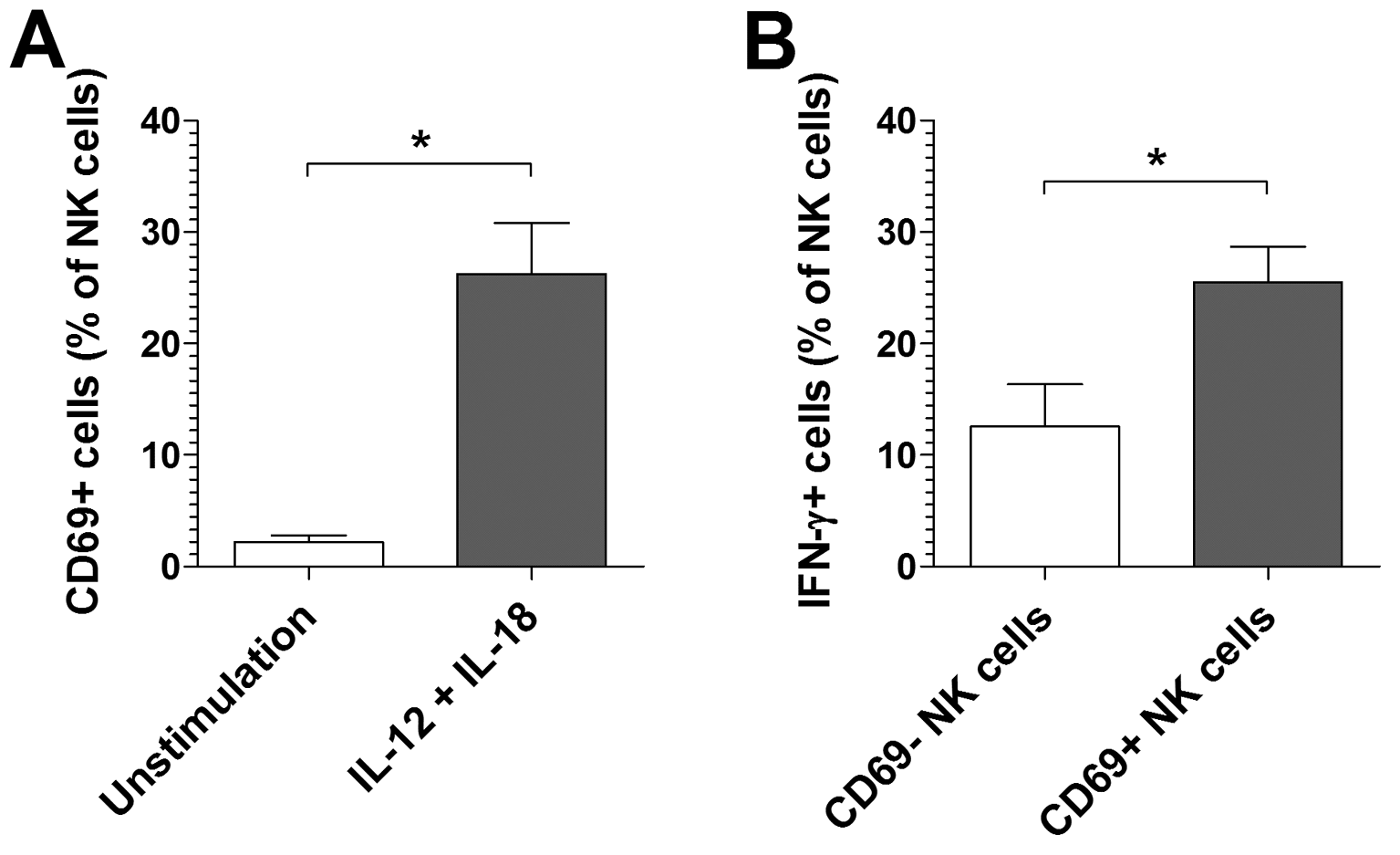

Supplement: S1 Fig — Freshly isolated PBMCs (1 × 10^6^/well) were incubated for 24 hours in the presence of IL-12 (50 ng/mL) and IL-18 (50 ng/mL), or PBS as a control. Expression levels of CD69 and IFN-γ in NK cells were determined by flow cytometry after stimulation with IL-12 and IL-18. Panel A: CD69 expression in NK cells derived from 3 independent experiments using HC as determined by flow cytometry. Panel B: IFN-γ-expressing cells in CD69+ and CD69- NK cell subsets derived from 3 independent experiments using HC as determined by intracellular flow cytometry. Values are expressed as the mean ± SEM. *p < 0.05 by paired t-test. (TIF) [file pntd.0005815.s004.tif]

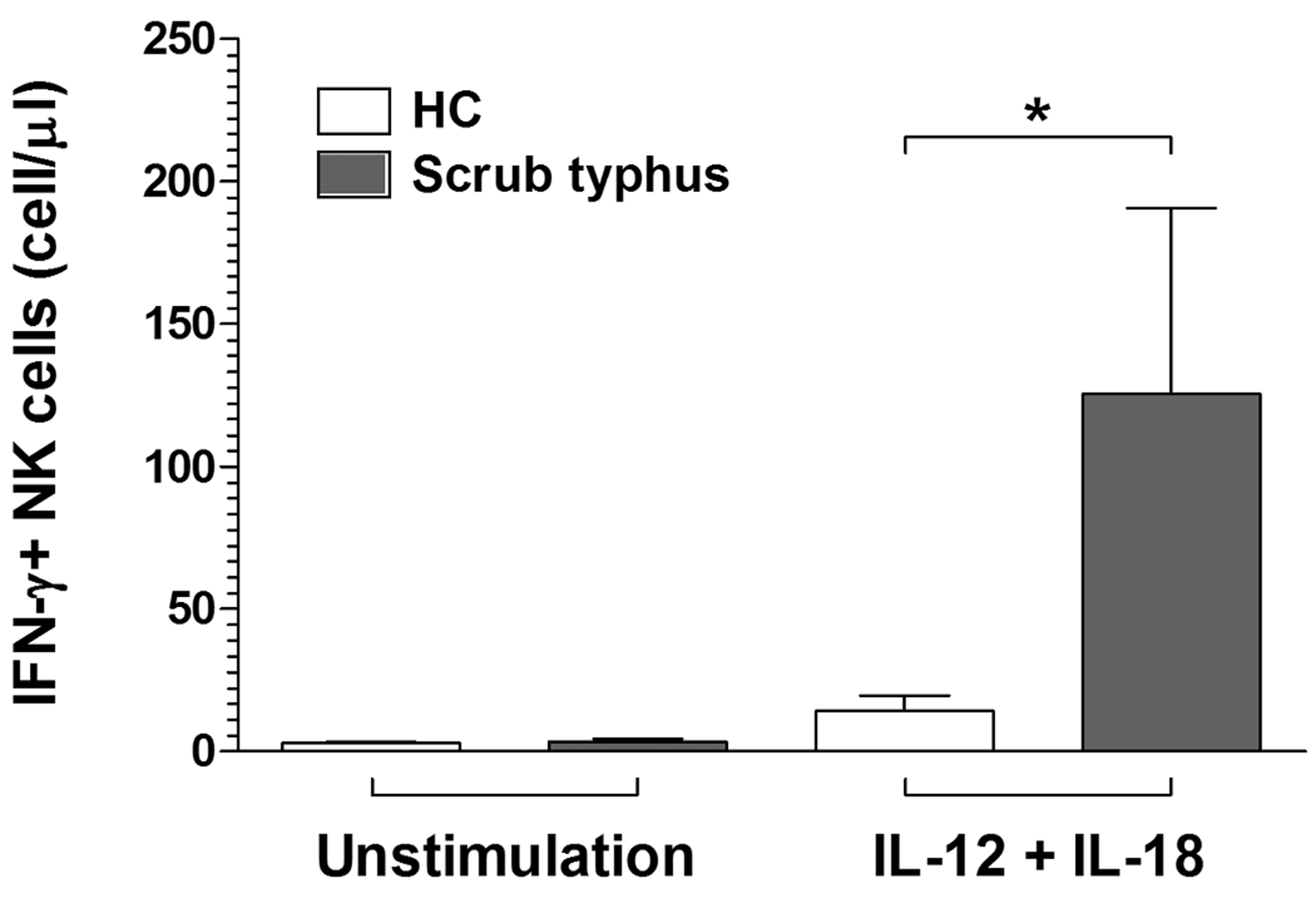

Supplement: S2 Fig — Freshly isolated PBMCs (1 × 10^6^/well) were incubated for 24 hours in the presence of IL-12 (50 ng/mL) and IL-18 (50 ng/mL), or PBS as a control. Percentages of total and IFN-γ+ NK cells were determine by flow cytometry among lymphocytes and total NK cell proportion. Absolute IFN-γ+ NK cell numbers were calculated by multiplying NK cell and IFN-γ+ NK cell percentages by total lymphocyte numbers (per microliter). Data were obtained from 10 HCs and 5 patients with scrub typhus. Values are expressed as the mean ± SEM. *p < 0.05 by unpaired t-test. (TIF) [file pntd.0005815.s005.tif]

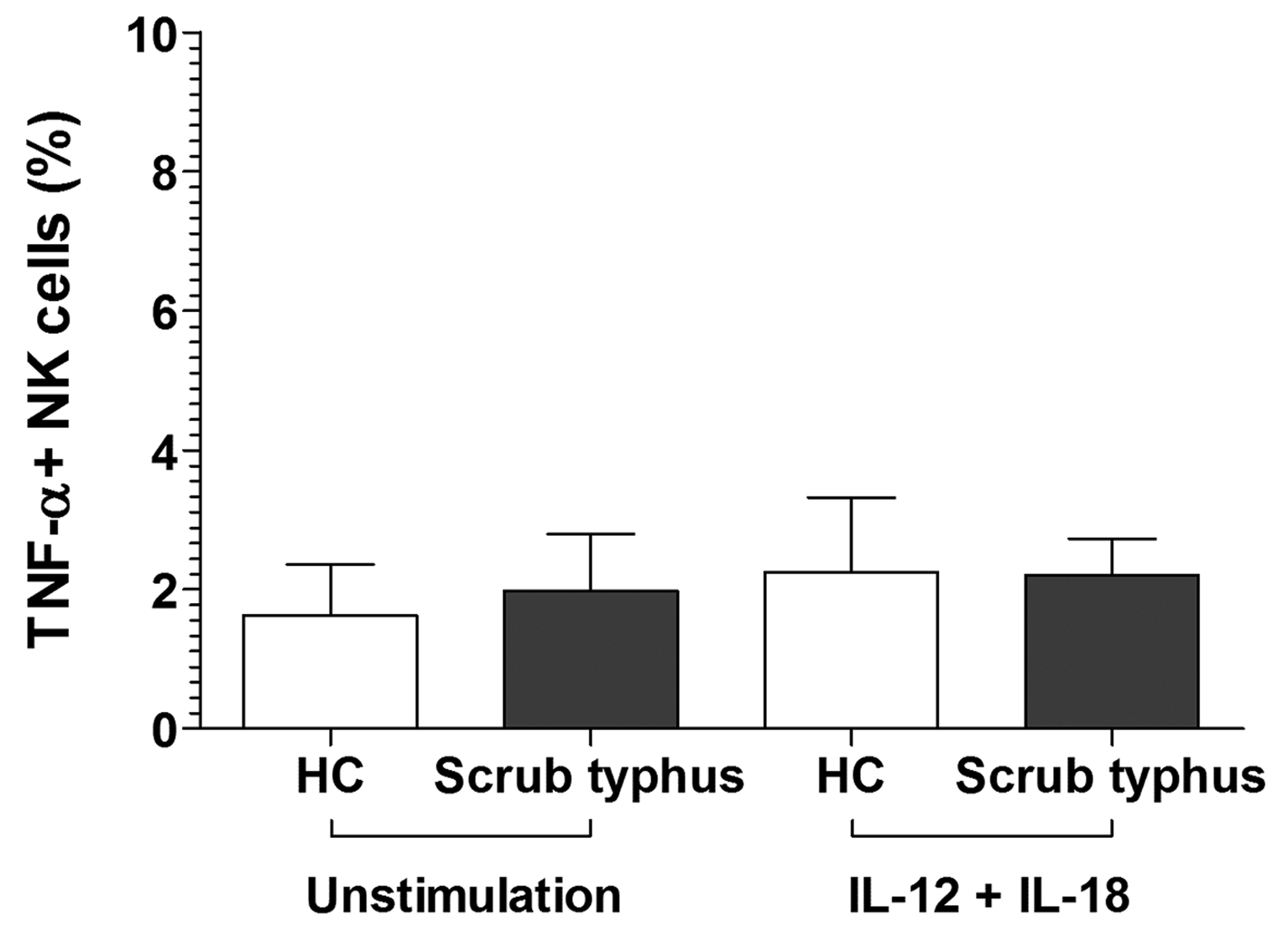

Supplement: S3 Fig — PBMCs (1 × 10^6^/well) were incubated for 24 hours in the presence of IL-12 (50 ng/mL) and IL-18 (50 ng/mL), or PBS as a control. Expressions of TNF-α in NK cells were determine by flow cytometry after stimulation with IL-12 and IL-18. Percentage of TNF-α-expressing cells in total NK cells as determined by intracellular flow cytometry. Data were obtained from 10 HCs and 5 scrub typhus patients. Values are expressed as the mean ± SEM. An unpaired t-test was used to compare expression levels of TNF-α in scrub typhus patients versus healthy controls. (TIF) [file pntd.0005815.s006.tif]

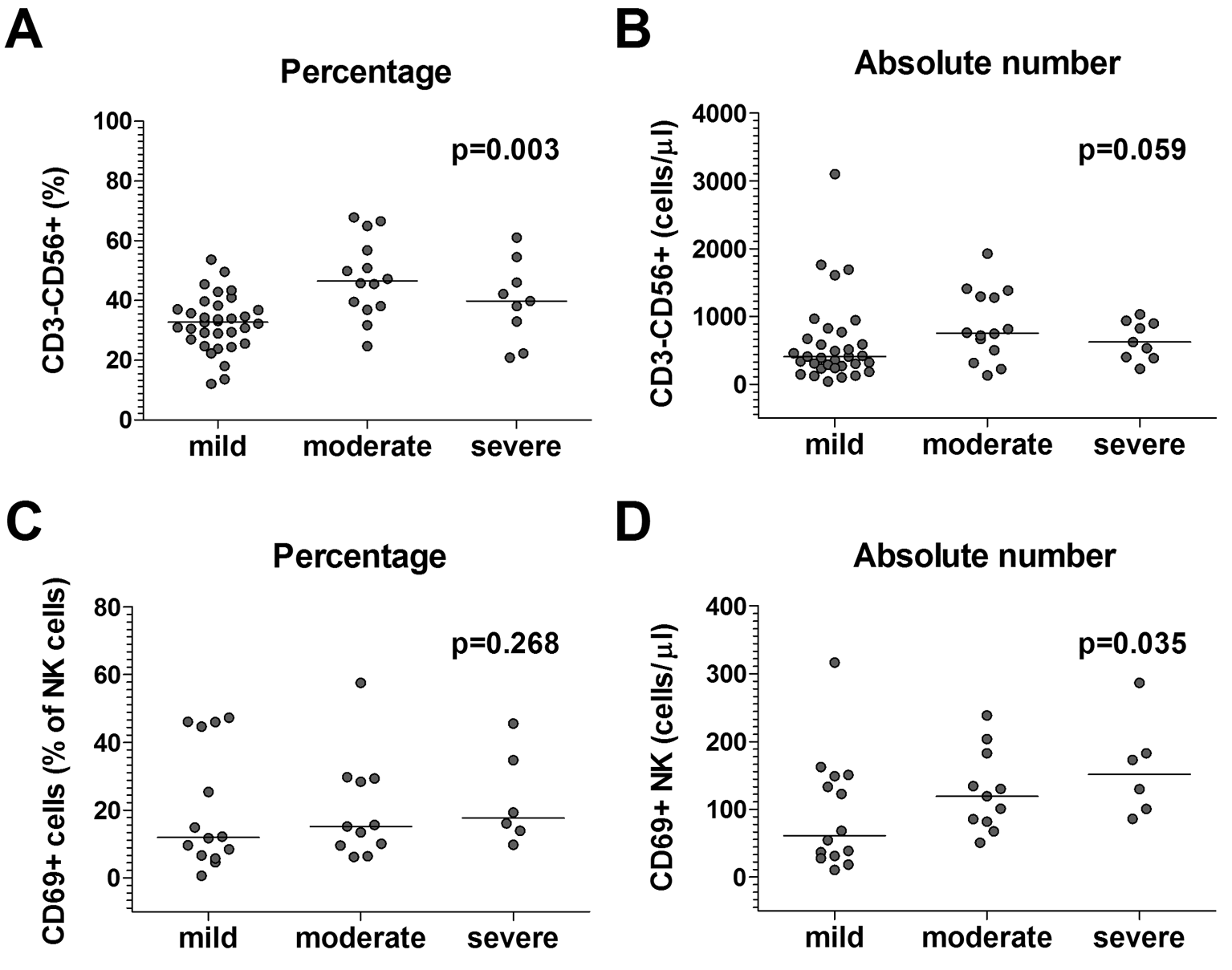

Supplement: S4 Fig — Freshly isolated PBMCs from patients with scrub typhus were stained with either a cocktail of FITC-conjugated anti-CD56, PE-conjugated anti-CD3, and PerCP-conjugated anti-CD45 mAbs or another cocktail of FITC-conjugated anti-CD56, PE-conjugated anti-CD69, APC-conjugated anti-CD3, and PerCP-conjugated anti-CD45 mAbs, and then analyzed by flow cytometry. According to the disease severity, scrub typhus are classified into mild, moderate, and severe disease. Panel A and B: Percentages (among peripheral blood lymphocytes) and absolute numbers (per microliter of blood) of total NK cell population for a total of 56 patients with scrub typhus. Panel C and D: Percentages (among total NK cells) and absolute numbers (per microliter of blood) of CD69+ NK cell population for a total of 31 patients with scrub typhus. Symbols represent individual subjects and horizontal lines indicate median values. The p values for the trend of NK cell levels according to the disease severity were calculated by the Jonckheere-Terpstra test. (TIF) [file pntd.0005815.s007.tif]
